# Training in robotic surgery: initial experience using the Brazilian College of Surgeons model

**DOI:** 10.1590/0100-6991e-20202969

**Published:** 2021-06-04

**Authors:** FERNANDO DE BARROS, VERONICA BERNARDINO FELICIO, ANA CAROLINE LIMA TABET, ANA CAROLINA CAPUANO CERBONE

**Affiliations:** 1 - Universidade Federal Fluminense, Departamento de Cirurgia Geral e Especializada - Niterói - RJ - Brasil; 2 - Hospital São Lucas, Departamento de Cirurgia Geral - Rio de Janeiro - RJ - Brasil

**Keywords:** Robotic Telesurgery, Training, Robotic Surgical Procedure, Telecirurgia Robótica, Treinamento, Procedimento Cirúrgico Robótico

## Abstract

**Objective:**

to present the initial experience of the first tier of surgeons trained in the new model of robotic surgery training proposed by the CBC.

**Methods:**

we retrospectively collected data and information on training with the Da Vinci SI robotic system. The variables analyzed were, in the pre-clinical phase, time of completion of each step by surgeon and number of hours in the simulator, and in the clinical phase, operations carried out by the training group, number of surgeons who performed nine procedures in ninety days (“9 in 90”), time of docking, time of console, and results surgical*.*

**Results:**

we interviewed 39 surgeons before training started; 20 (51.3%) reached the clinical phase. The average age of surgeons was 47.9 years (38-62). The average time between the first interview and the delivery of the online certificate was 64 days (15-133). The surgeons have made an average of 51h and 36 minutes of robot simulation (40-83 hours). The total number of cases in which the training surgeons participated as first assistant was 418, with an average of 20.9 per surgeon. The time of pre-clinical training had an average of 116 days (48-205).

**Conclusion:**

the new model proposed had good acceptance by all surgeons trained and proved safe in the initial sample.

## RESUMO

**Objetivo:**

apresentamos nesse artigo o resultado da experiência inicial do nosso programa durante o treinamento dos primeiros cirurgiões no novo modelo de treinamento em cirurgia robótica proposto pelo CBC.

**Métodos:**

avaliamos retrospectivamente por coleta de dados e informações sobre treinamento no sistema robótico Da Vinci SI. As variáveis analisadas foram: Fase Pré-clínica: tempo de conclusão de cada uma das etapas por cirurgião, número de horas no simulador; Fase clínica: operações realizadas pelo grupo em treinamento, número de cirurgiões que realizaram nove procedimentos em noventa dias (“9 em 90”), tempo de docking, tempo de console, resultados cirúrgicos.

**Resultados:**

trinta e nove cirurgiões foram entrevistados para início do treinamento, 20 (51,3%) chegaram à fase clínica. A média de idade dos cirurgiões foi de 47,9 (38 a 62) anos. O tempo médio entre a primeira entrevista e a entrega do certificado online foi de 64 dias (15 a 133). Os cirurgiões fizeram média de 51h e 36 minutos de simulação robótica (40 a 83 minutos). O número total de casos em que os cirurgiões em treinamento participaram do ato cirúrgico como primeiro assistente foi de 418 casos, com média de 20,9 por cirurgião. O tempo de treinamento pré-clínico teve média de 116 dias (48 a 205 dias).

**Conclusão:**

o novo modelo proposto teve boa aceitação por todos os cirurgiões treinados e se mostrou seguro na amostra inicial.

## INTRODUCTION

Robotic surgery has been initially accomplished with platforms used for neurosurgical biopsies, called Programmable Universal Machine for Assembly (PUMA) 200^1^. Since then, many platforms evolved and, in 1998, Computer Motion introduced the Zeus system, in which the surgeon controls arms and instruments^2^. However, the greater impact on the evolution of robotic surgery was when Intuitive Surgical developed the Da Vinci platform, in 1998.

Since then, the robotic surgery has been growing exponentially, as well as the number of surgeons adopting the method. This demand eventually created the need for training of surgeons to operate the machine safely^3^. However, the certification process had been being carried out worldwide by the company which owns the Da Vinci platform^4^.

In 2020, in an unprecedented action, the Commission of Minimally Invasive and Robotic Surgery of the Brazilian College of Surgeons (CBC) lunched an initiative for the establishment of a certification, based on objectives and validated criteria, for the conduction of robotic procedures, based on a document of the Brazilian Medical Association^5^. Essentially, training becomes a responsibility of each hospital, divided into four steps: 1) Introduction to robotic system; 2) Theoretical-practical training on the robotic platform; 3) Pre-clinical training; 4) Clinical training under mentoring.

## OBJECTIVE

We present the initial experience of our program during the first year of training, of the first surgeons tier, with this new model of training in robotic surgery proposed by the CBC.

## METHODS

The study was submitted to the Ethics in Research Committee (CAEE 67889617.3.0000.5533) and approved by Opinion 2,200,788 of CEP, Pró-Cardíaco-ESHO Empresa de Serviços Hospitalares. The period of recruitment (interview) was from January to December 2020 in a continuously flow in the program. Since this work is only descriptive, we use sampling by convenience. We retrospectively collected data in the São Lucas Copacabana hospital system on the initial experience with the new surgeons trained in the model recommended by the CBC on the robotic platform Da Vinci SI. All surgeons trained met the following criteria: professional qualification and specialty (RQE) in any surgical area; minimum experience of five years in the respective specialty; and be duly registered at the hospital specialty service.

**Figure 1:**
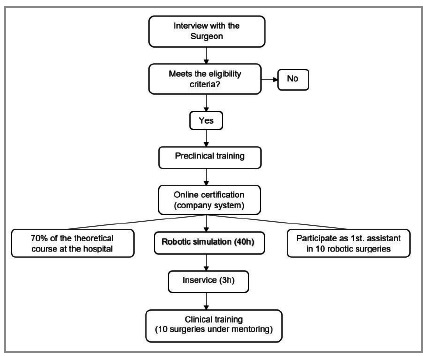
Training algorithm.

Figure 1 summarizes the training flow. After the interview with the coordination of the robotic surgery program, the first step required of the surgeon in the pre-clinical phase was the conclusion of the online course at the Intuitive site that introduces the robotic system. After the delivery of the certificate, the surgeon was introduced to the simulator (Simbionix -Endocompany®) along with the robotic assisting nurse, previously trained and qualified in the simulator. Scheduling of the simulator was freely opened, with total autonomy for the surgeon to schedule training sessions. We considered the simulation phase completed when the surgeon reached 40 hours, with at least 80% proficiency in all simulator exercises, among these, camera navigation, clutching, setup of the fourth arm, delivery of energy, manipulation of the endowrist, suturing, perception of depth, and manual dexterity. There was no training with live tissue or use of animals in the laboratory.

During the simulation phase, the surgeon was encouraged to perform 10 cases as the first assistant for robotic surgeries performed at the hospital. All participated in the robotic surgery theoretical course (from basics to specialty), with minimum eight hours evaluation time. To pass to the next training step, the surgeon needed a 70% grade in the theoretical course. During this period, we offered a collection of robotic procedures via internet and encouraged the trainees to attend at least five procedures of their specialty.

For theoretical and practical training in the robotic platform (Inservice), the surgeon ought to complete all the previous steps and have at least two operations scheduled within two weeks. During the Inservice phase, all were trained to master the operating room setup, system setup, robotic drape placement, movement of the patient robotic cart, port placement, docking, solving any issues with the platform, and predetermined exercises: third arm movement, suturing, and camera movement. The Inservice phase lasted three hours for each surgeon.

We expressed continuous variables as average and range, those being time of completion of each of the steps by the surgeon, number of hours needed to complete the proficiency in the simulator, total number of operations carried out by the training group, number of surgeons who performed nine procedures in ninety days (“9 in 90” - goal strategy proposed by Intuitive), time of docking, time of console, surgical results, and number of surgeons with complete mentored clinical training.

## RESULTS

Although the recruitment period was between January the December 2020, all operations were performed in the period from March to December 2020 due to uncontrollable reasons at the time of the study (pandemic by SARS COV2). We interviewed 39 surgeons for the start of training, 20 of them (51.3%) having reached the clinical phase, and among them, five (25%) have completed it and, as of this writing, operate without the need of a preceptor. The average age among surgeons was 47.9 years (range 38 62) (Table 1). The average time between the first interview and the delivery of the online certificate was 64 days (range 15-133). As for the simulator, the surgeons performed an average of 51 hours and 36 minutes (range 40 83 hours) of simulation. The average time to reach 80% skill proficiency in the exercise was 29 hours and 34 minutes (range 13-35 hours) of simulation. Sixteen (80%) surgeons reached the required proficiency in less than 40 hours. The total number of cases in which the training surgeons participated was 418, with an average of 20.9 cases per surgeon. The average time of pre-clinical training was 116 days (range 48-205). Among the 124 operations performed, 13 (10.5%) took place without a preceptor.

**Table 1:** Characteristics of surgeries performed.

Surgery	n	Surgery time (min)			Docking time (min)		
		Average	Variation	SD	Average	Variation	SD
Gastroplasty	76	123.6	65-210	30.5	7.5	3.0 - 14.2	2.3
Inguinal herniorrhaphy	13	144.0	75-230	48.9	5.2	3.4 - 6.8	0.9
Partial Colectomy	10	132.5	130-135	3.5	9.1	6.11 - 11.2	1.8
Hysterectomy	6	153.8	68-215	51.9	4.9	3.2 - 6.5	1.5
Reflux	6	134.2	65-225	58.7	8.0	6.6 - 8.7	0.8
Cholecystectomy	3	83.0	65-101	25.5	5.8	5.4 - 7.2	1.1
Prostatectomy	3	263.3	240-305	36.2	8.3	7.5 - 9.7	1.2
Duodenopancreatectomy	2	385.5	337-380	30.4	13.4	12.0 - 14.4	1.6
Pulmonary Lobectomy	2	215.0	190-240	35.4	17.2	16.7 - 17.7	0.7
Esophagomyotomy	1						
Mediastinal lymphadenectomy	1						
Nephrectomy	1						

SD - Standard Deviation.

Table 2 brings the records of the number and the characteristics of each surgical procedure. The age of the patients operated was of 43.5 ± 13.3 years (range 21-88). The most commonly conducted procedure was gastroplasty, with 76 cases (61.3%).

**Table 2:** Stages of the Pre-clinical and Clinical phases.

Surgeon	Age (years)	Online Certificate Delivery Time (Days)	Simulation time to achieve 40h (Days)	Surgeries as First Assistant	Preclinical Training Time	Number of surgeries	Under Mentoring	Without Mentoring
1	54	95	65	10	124	18	10	8
2	56	105	14	16	158	12	10	2
3	38	42	56	21	118	11	10	1
4	41	41	52	160	117	11	10	1
5	62	15	91	10	205	11	10	1
6	39	46	27	10	56	9	9	0
7	46	65	17	31	148	9	9	0
8	47	73	60	10	190	8	8	0
9	41	63	63	10	105	6	6	0
10	39	62	18	18	100	5	5	0
11	40	44	47	10	99	4	4	0
12	62	133	36	10	148	4	4	0
13	54	28	52	19	182	3	3	0
14	52	81	20	10	54	3	3	0
15	53	32	26	12	54	3	3	0
16	48	93	33	10	48	2	2	0
17	56	117	91	10	154	2	2	0
18	42	81	80	10	121	1	1	0
19	38	36	51	10	60	1	1	0
20	51	37	55	21	71	1	1	0
TOTAL				418		124	111	13

There was no unforeseen event or accident with the robotic platform. There was only one death, a patient who was subjected to pancreatoduodenectomy and had major bleeding on the 16th postoperative day. The average time of hospitalization was 2.02 days (range 1-21). At the time of the study conclusion, among the 10 surgeons who had more than 90 days since the first case, six (60%) reached the proposed target of nine procedures in 90 days.

## DISCUSSION

Few protocols for training in robotic surgery were validated by means of work reviewed by peers. In addition, there is a lack of information on the characteristics of surgeons and training in robotic surgery that impact the surgeon’s performance on the console^6^. As far as we know, in March 2020, our group held the first operations with the first robotic surgeons trained with this new model designed by the CBC. The idea of this work was to report the first results of the first year, to make a critical analysis, and to convey the impression of the initial clinical practice.

There is no doubt about the value of the robotic platform for the evolution of surgery. However, surgeons there are not qualified, are poorly trained, or even are poorly oriented, can put their patients at risk^7^. The regulated training offering is fundamental, as well as naming the ones technically responsible the robotic procedure (surgeon, preceptor, and the institution, represented by its technical director). The new rules, with oversight of assignments, responsibility, and training by the institution, with guidance and regulation of medical societies, seem to have good acceptance among the surgeons of the country, as already published^5^ ^,^ ^8^. 

The experience gained in recent years in established programs can no doubt influence the outcome of this new training model. The first point to be discussed is the completion of the pre-clinical phase by little more than half of the initially enrolled surgeons. We believe that the pandemic has in some way interfered with the adherence of some surgeons, who preferred not to go to the hospital in the study period. Besides that, despite the interest initially shown by many, these could not manage to adapt to the day to day training, which in fact influences the surgeon’s performance and can be deterring during the training programs^9^. 

The time between the interview with the coordinator and the delivery of the online certificate was on average of 64 days. This is long, reflected in the great difficulty of the surgeons with the current assessment offered on the company’s site. This assessment is passing by constant changes and, in our opinion, need to be revised, since many issues addressed are of engineering technical order and non surgical.

As long-established, simulation and repetition in surgery are critical^10^. The training in robotic surgery simulation is without doubt a great technical education tool for new technologies in surgery^11^. The time of simulation proposed to surgeon was 40 hours, with a minimum of 80% proficiency. The great majority (80%) achieved proficiency with less than 40h, what we deemed sufficient for the next training steps - Inservice and the clinical phases. We believe that training in animals can be useful, however not necessarily mandatory, in accordance with the new resolution^5^. None of the training surgeons studied trained in animals.

It is noteworthy the number of robotic procedures with the involvement of surgeons, 418 for 20 surgeons, averaging 20.9 operations per surgeon. In fact, three of the trained surgeons already worked in teams with large volumes of robotic surgery, what caused the boosted average. Together, the three participants operated on 212 cases (50.7%). Clearly, these surgeons got easily through the clinical training phase (port placement and docking). Nonetheless, in general 10 cases were enough for every surgeon to feel comfortable with the Da Vinci Si platform and thus pass to the clinical training phase. As Zhao et al. reported, the progression of the first assistant in the field to the console is fundamental and necessary, as it gives the trainee great confidence in handling the platform^12^. This phase of training (pre-clinical) had an average of 116 days and the longest part of this time was for the online certification, as mentioned.

Gastroplasty was the most common operation - 76 cases (61.3%). The profile of surgeons trained in this beginning of the new program was crucial for such a discrepancy as to other procedures. Besides that, even those surgeons who perform other types of operations chose gastroplasty, due to its good volume and standardization, two essential features in this initial training curve. This learning curve can be decreased according to several factors^13^. However, the volume of procedures is the main factor determining the learning curve, and this is clear for the training of the 10 (50%) surgeons with higher volume, who reached the “9 in 90” target. We should emphasize that we always encourage new training surgeons to choose operations they perform routinely, regardless of complexity. Nevertheless, Formisano et al. report that having or not experience in certain procedures by the conventional or laparoscopic methods may not be necessary^14^. 

We regarded the operations by this new group as if this were again the atarting point of a robotic surgery program. As well as in the inception of our program years ago, we had no accidents or complications with the robotic platform, which is in agreement with other studies published in the literature that describe this beginning^15^ ^-^ ^17^. The cause of death of the patient submitted to pancreaticoduodenectomy had no relation with problems or errors due to the robotic platform. On the 16th postoperative day, the patient had an extensive intra-abdominal bleeding after the apparent resolution of a biliary fistula that lasted for 10 days. The patient was in the ward with scheduled discharge and ended up being subjected to an emergency laparotomy that showed a lesion of the dorsal pancreatic artery, probably caused by the corrosion of the vessel by the biliary secretion.

As a major limitation of the study, we acknowledge the lack of statistical calculation of the sample, so that these results could be representative of the population of surgeons who perform this training. As the study was descriptive, we used a convenience, non-probabilistic, non-random sample. The main idea was to describe the initial experience of the first year of training in robotic surgery using the model created by the Brazilian College of Surgeons.

## CONCLUSION

Our initial sample of surgeons submitted to the new training model proposed by the CBC had good acceptance and safety in the first training year.
